# Triangular fossa of the third cerebral ventricle – an original 3D model and morphometric study

**DOI:** 10.3389/fnana.2024.1398858

**Published:** 2024-07-29

**Authors:** Alin Horatiu Nedelcu, Vasile Valeriu Lupu, Ancuta Lupu, Razvan Tudor Tepordei, Ileana Ioniuc, Cristinel Ionel Stan, Simona Alice Partene Vicoleanu, Ana Maria Haliciu, Gabriel Statescu, Manuela Ursaru, Ciprian Danielescu, Cristina Claudia Tarniceriu

**Affiliations:** ^1^Department of Morpho-Functional Science I, Discipline of Anatomy, “Grigore T. Popa” University of Medicine and Pharmacy, Iasi, Romania; ^2^Department of Mother and Child, “Grigore T. Popa” University of Medicine and Pharmacy, Iasi, Romania; ^3^Department of Surgical Sciences I, “Grigore T. Popa” University of Medicine and Pharmacy, Iasi, Romania

**Keywords:** triangular recess, ventriculoscopy, anterior commissure, vestibulum, vulva cerebri, endoventricular navigation, Schwalbe’s fossa, third ventricle

## Abstract

**Introduction:**

The triangular recess (TR), also called triangular fossa or *vulva cerebri,* represents the anterior extension of the diencephalic ventricle, located between the anterior columns of the fornix and the anterior white commissure. Over time, this structure of the third cerebral ventricle generated many disputes. While some anatomists support its presence, others have opposite opinions, considering that it only becomes visible under certain conditions. The aim of the study is to demonstrate the tangible structure of the triangular recess. Secondly, the quantitative analysis allowed us to establish an anatomical morphometric standard, as well as the deviations from the standard.

**Materials and methods:**

Our study is both a quantitative and a qualitative evaluation of the triangular fossa. We dissected 100 non-neurological adult brains, which were fixed in 10% formaldehyde solution for 10 weeks. The samples are part of the collection of the Institute of Anatomy, “Grigore T. Popa” University of Medicine and Pharmacy, Iasi. We highlighted the triangular fossa by performing dissections in two stages at the level of the roof of the III ventricle.

**Results:**

The qualitative analysis is a re-evaluation of the classical data concerning the anatomy of the fossa triangularis. We proposed an original 3D model of the triangular recess in which we described a superficial part called vestibule and a deep part called *pars profunda*. We measured the sides of the communication between the two proposed segments, as well as the communication with the III ventricle. By applying the Heron’s formula, we calculated the area of the two communications. Statistical evaluations have shown that these communications are higher than they are wide. In addition, there is a statistical difference between the surfaces of the two communications: 34.07 mm^2^ ± 7.01 vs. 271.43 mm^2^ ± 46.36 (*p* = 0.001).

**Conclusion:**

The outcome of our study is both qualitative and quantitative. Firstly, we demonstrated the existence of the triangular fossa and we conceived a spatial division of this structure. Secondly, the measurements carried out establish an anatomo-morphometric norm of the triangular recess, which is useful in assessing the degree of hydrocephalus during the third endoscopic ventriculoscopy.

## 1 Introduction

The triangular fossa is a diverticulum of the third cerebral ventricle. It lies on the anterior wall between the anterior columns of the fornix and the anterior white commissure. The *lamina terminalis* delimits anteriorly this recess (Fernandes-Silva et al., 2021; Abdala-Vargas et al., 2022; Theologou et al., 2023).

The development of endoscopic neurosurgery techniques requires a more accurate knowledge of the ventricular anatomy. In this context, the anterior wall of the third cerebral ventricle acquires a crucial importance, having both landmark value and approach virtue. The anterior transcortical, transcallosal interhemispheric, transsphenoidal translaminar and subfrontal translaminar surgical approach techniques used during ventriculoscopy must consider its anatomy, as well as the numerous anatomical variants of the anterior cerebral artery and its branches. Among these, it is worth mentioning that duplications and fenestrations are frequent anomalies that can put in difficulty even the most experienced neurosurgeons (Schroeder et al., 2002; Makowicz et al., 2013; Nedelcu et al., 2016; Dumitrescu et al., 2022; Dang et al., 2023; Majmundar et al., 2023).

Over time, the triangular recess has raised numerous discussions. Some anatomists support its existence while others dispute it. A third category states that it is a virtual structure, which becomes a real space in certain conditions such as hydrocephalus (Longatti et al., 2003; Feletti et al., 2016).

The aim of our study is to clearly demonstrate the existence of the triangular recess and to show that, under the magnifying instruments, the fossa is not a simple vertical recess, but has a complex structure. Consequently, we performed a morphometric study of the triangular recess aperture and we proposed an original 3D model of this fossa.

## 2 Materials and methods

We dissected 100 adult brains with no known neurological abnormalities, which were fixed in 10% formaldehyde solution for a minimum of 10 weeks. We dissected the brains under an OPTRON—Zeiss OPM 6 operator microscope at the Institute of Anatomy, “Grigore T. Popa” University of Medicine and Pharmacy, Iasi. We used a Nikon D7000 digital camera equipped with a 60 mm AF Micro-Nikkor f/2.8D lens to acquire the images and processed them with Adobe Photoshop CS5 and Capture NX2 software.

**Figure 1:**
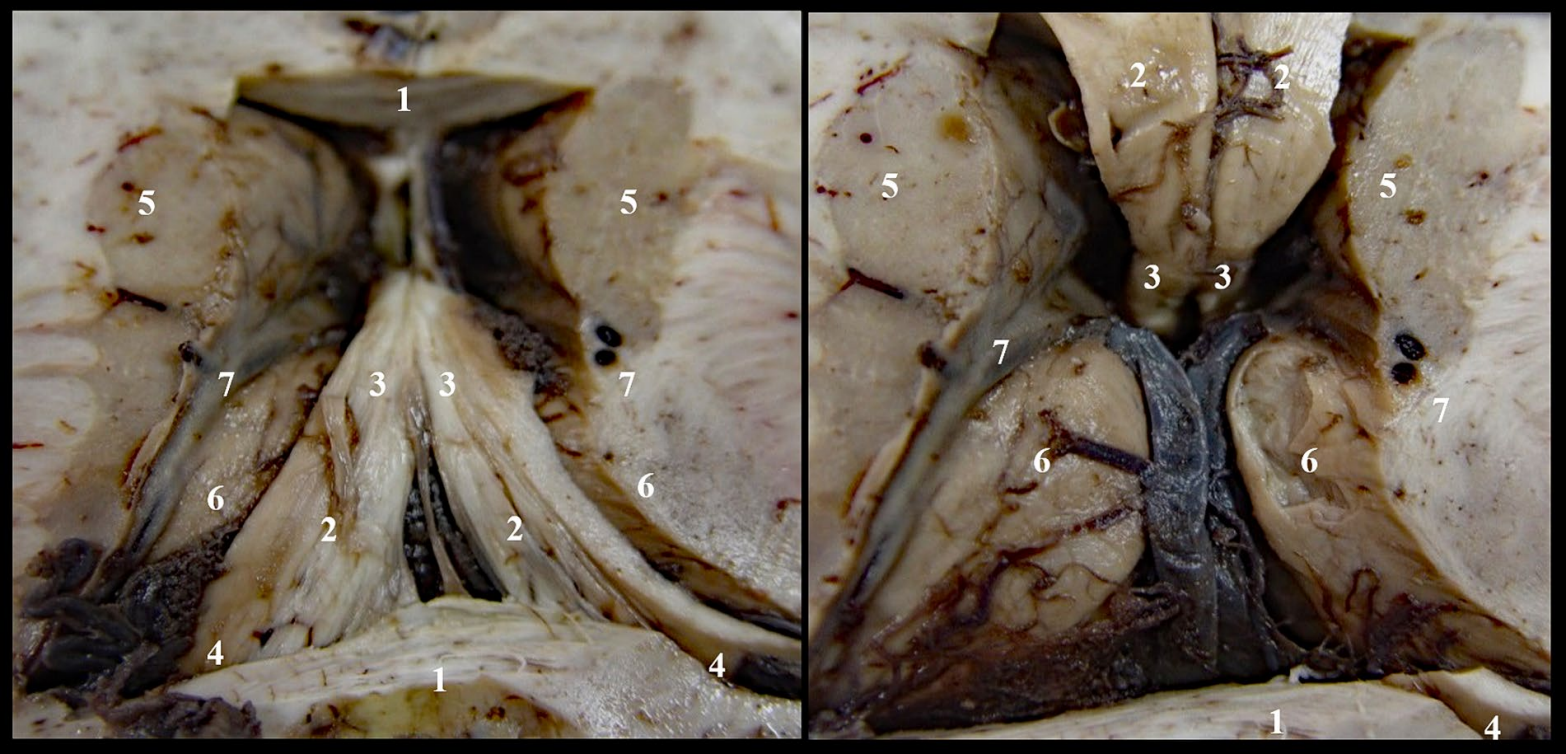
Denudation of third ventricle’s roof, stage I: 1. Corpus callosum; 2. Body of fornix; 3. Anterior pillars of the fornix; 4. Posterior pillars of the fornix; 5. Caudate nucleus; 6. Thalamic nuclei; 7. Optostriat sulcus and vein, lamina affixa and tenia semicircularis.

**Figure 2:**
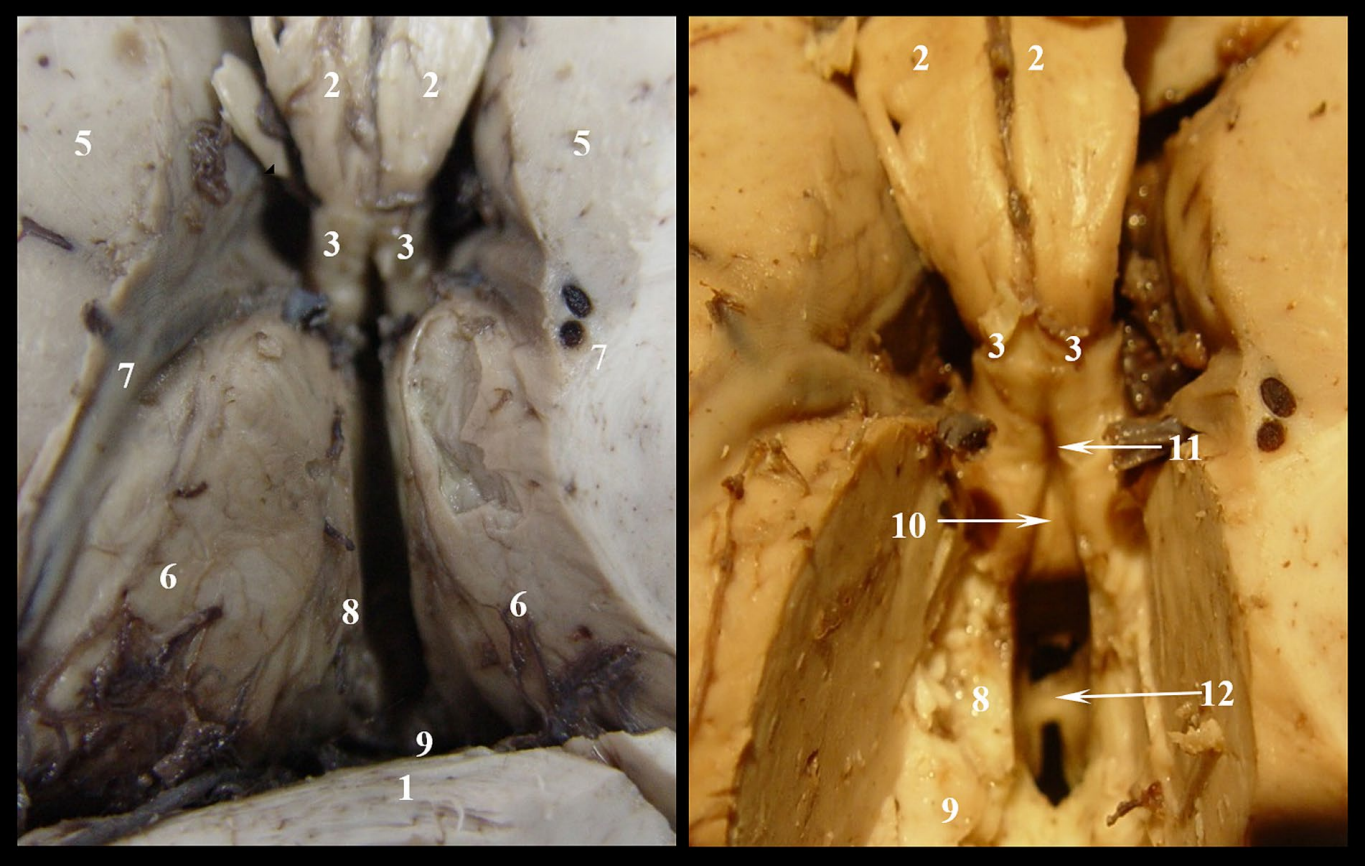
Denudation of third ventricle’s roof, stage II: 1. Corpus callosum; 2. Body of fornix; 3. Anterior pillars of the fornix; 4. Posterior pillars of the fornix; 5. Caudat nucleus; 6. Thalamic nuclei; 7. Optostriat sulcus and vein, lamina affixa and tenia semicircularis; 8. Tenia thalami (habena); 9. Epiphiseal recessus; 10. Comissura rostralis; 11. Fossa triangularis; 12. Interthalamic adhesion.

We denudated each layer to reach the roof of the third ventricle and to identify the superior surface of the thalamic nuclei, while keeping intact the cerebral veins and the choroid plexus (Figure 1). In the second stage of dissection, we exposed the triangular fossa by removing the supero-medial fourth of the thalamic nuclei (Figure 2).

We studied the morphological aspect and we measured the parameters of the triangular recess by using a millimeter scale and the software previously mentioned; we calculated the communicating area with the third ventricle using the Heron’s formula. The obtained data was statistically processed by using the interval of variation method, Pearson correlation and Paired Samples T-test.

The study of the correlation between different phenomena was carried out using the correlation coefficient “r” (Pearson). It shows the intensity of the statistical links, as well as their direction. The correlation coefficient values are between [−1, +1]: A coefficient of +1 indicates that the two variables are perfectly positively correlated, so that if one variable increases, the other increases by a proportionate amount. Conversely, a coefficient of −1 indicates a perfect negative relationship: if one variable increases, the other decreases by a proportionate amount. If the correlation coefficient is 0, there is no association or link between the two variables.

## 3 Results

In our concept, the triangular recess has a complex morphological structure, and we propose a spatial model of organization in which the fossa consists of two parts: one deep and one superficial.

### 3.1 The deep part (pars profunda) of the triangular recess

**Figure 3:**
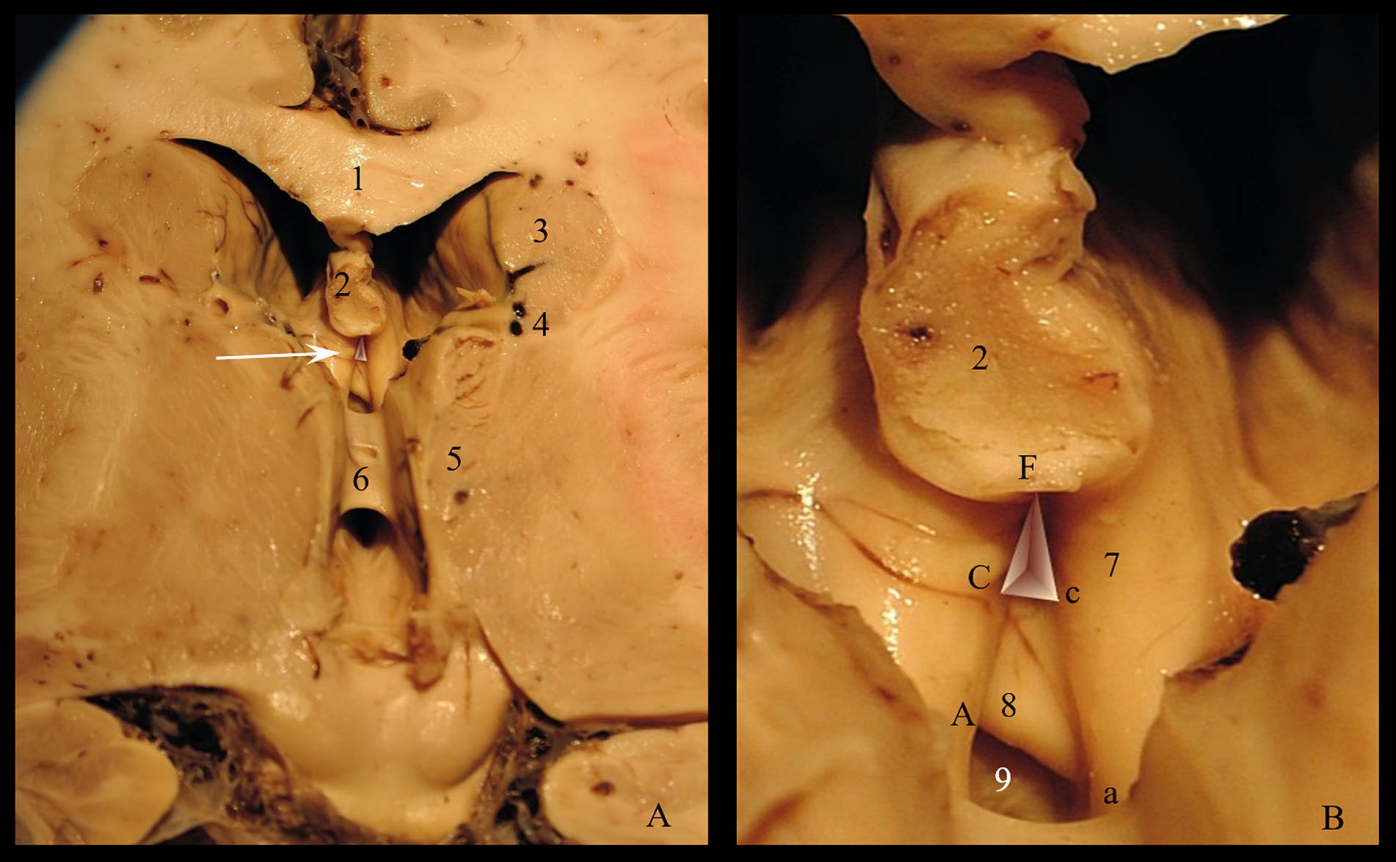
Triangular recess: **(A)** Roof of the third ventricle (arrow indicates the Triangular recess) **(B)** Morphometric parameters of the triangular recess. Pars profunda: Cc = the base; FC = left wall; Fc = right wall; Pars superficialis: Aa = the base; FA = left wall; Fa = right wall. 1. Corpus callosum; 2. Fornix; 3. Caudate nucleus; 4. Optostriat sulcus; 5. Thalamus; 6. Interthalamic adhesion; 7. Anterior crus of fornix; 8. Commissura rostralis; 9. Recessus subcommissuralis and *lamina terminalis*.

It has a quadrangular pyramidal shape that shows the following features (Figure 3):

- an anterior wall formed by the *lamina terminalis* that extends from the rostrum of the corpus callosum to the superior edge of the optic chiasm. We propose dividing it into three segments: the upper segment, called the recessus segment by us, which closes anteriorly the cavity of the fossa; the intermediate segment which is tangent to the anterior surface of the commissura rostralis, and the lower segment which stretches between the anterior surface of the rostral commissure and the anterior surface of the optic chiasm.
- lateral walls formed by the medial aspects of the anterior columns of the fornix in the segment between the tip of the fornix and the anterior white commissure.
- a posterior wall limited laterally by the two crura of the fornix and inferiorly by the commissura rostralis. Through this space, the triangular recess communicates with the superficial part, and we have named it ventricular aperture.
- a base corresponding to the upper surface of the commissura rostralis.
- a tip formed by the convergence of the two pillars of the fornix.

Since it represents the entry gate into the triangular fossa and, more than that, it is a ventriculoscopy landmark, we measured the limits of the posterior wall: the length of the inferior margin (anterior commissure-Cc), the length of the two lateral walls (left fornix-FC & right fornix-Fc) and we calculated the area of the ventricular aperture. The statistically processed results are shown in Table 1.

**Table 1:** Pars profunda.

		CC	Fc	FC	Posterior wall
N	Valid	100	100	100	100
	Missing	0	0	0	0
Mean		3.54	3.72^a^	3.75^a^	5.80
Median		3.57	3.71	3.75	5.86
Std. deviation		0.37	0.11	0.11	0.62
Variance		0.139	0.012	0.012	0.38
Skewness		−0.668	0.212	0.369	−0.455
Std. error of skewness		0.241	0.241	0.241	0.241
Minimum		2.44	3.51	3.54	4.09
Maximum		4.32	3.99	4.02	7.18
Percentiles	25	3.29	3.64	3.67	5.41
	50	3.57	3.71	3.75	5.86
	75	3.78	3.81	3.82	6.18

Descriptive statistics of triangular recess aperture (mm) and posterior wall (Heron’s formula). Paired Samples T test between triangular recess aperture ^a^*p* < 0.001.

The anterior commissure (Cc) varied from 2.44 to 4.32 mm, registering a mean level of 3.54 mm ± 0.37, which is close to the median value (3.57 mm). The right fornix-Fc varied from 3.51 to 3.99 mm, registering a mean level of 3.72 mm ± 0.11, which is close to the median value (3.71 mm). The left fornix-FC varied from 3.54 to 4.02 mm, recording a mean level of 3.75 mm ± 0.11, which is close to the median value (3.75 mm). The results of the Skewness test suggest the homogeneity of the range of values of Cc, Fc and FC. The mean level of Cc (3.54 mm) was significantly lower compared to the mean levels of Fc (3.72 mm; *p* = 0.001) or FC (3.75 mm; *p* = 0.001).

### 3.2 The superficial part (pars superficialis) of the triangular recess

**Figure 4:**
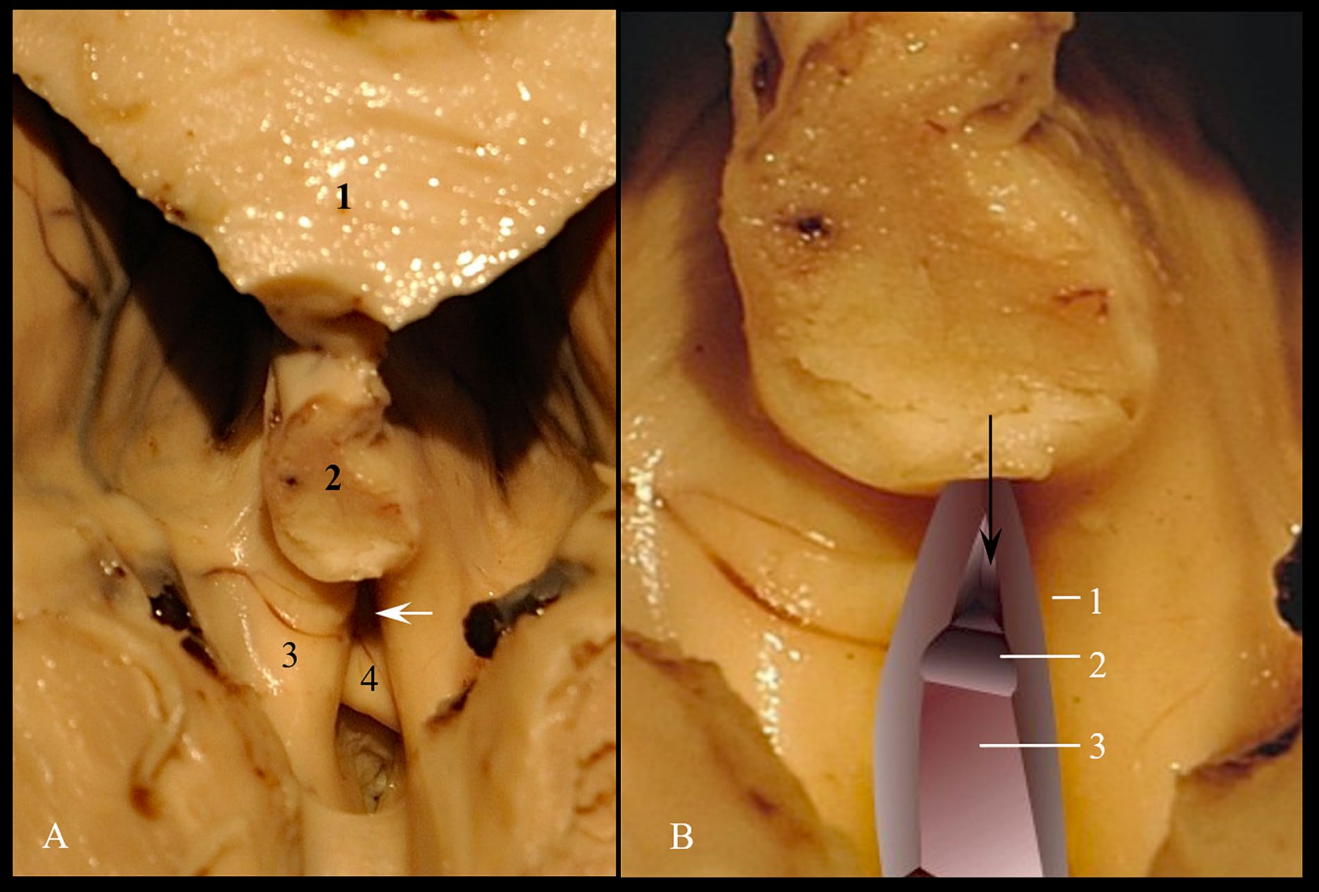
Triangular recess. **(A)** Anatomical landmark; 1. Corpus callosum; 2. Fornix; 3. Crus of fornix; 4. Commissura rostralis. **(B)** Spatial processing; 1. Medial wall of left crus of fornix; 2. Superior surface of commissura rostralis; 3. Retro-subcomisural area. The black arrow indicates the supracomisural segment of *lamina terminalis*. The white arrow indicates the entry into triangular recess.

It has a vestibular structure and is located behind the deep part, between the medial surfaces of the fornix cruses and the posterior surface of the anterior commissure. The medial surface of the post commissural pillars of the fornix forms the lateral walls. The base of the superficial part extends below the lower edge of the anterior commissure forming together with *lamina terminalis* a dead-end space, named by us retro-subcomisural area (Figure 4).

As for the deep part, we measured the limits of the posterior wall, which represents the passage to the cavity of the third cerebral ventricle. The posterior wall has a triangular shape. We measured the lower edge of the anterior white commissure included between the columns of the fornix (Aa), as well as the anterior columns of the fornix included between the tip of the fornix and the lower edge of the anterior white commissure (FA and Fa). We calculated the communication area between the vestibule and the ventricular cavity itself using Heron’s formula.

The anterior commissure (Aa) varied from 4.71 to 7.19 mm, recording a mean level of 6.09 mm ± 0.51, which is close to the median value (6.14 mm). The right fornix-(Fa) varied from 5.53 to 6.88 mm, recording a mean level of 6.19 mm ± 0.32, which is close to the median value (6.18 mm). The left fornix (FA) varied from 5.57 to 6.94 mm, recording a mean level of 6.24 mm ± 0.31, which is close to the median value (6.20 mm). The results of the Skewness test suggest the homogeneity of the values of Aa, Fa and FA. The average level of Aa (6.09 mm) was significantly lower compared to the average level of FA (6.24 mm; *p* = 0.011), but not compared to Fa (6.19 mm; *p* = 0.115) (Table 2).

**Table 2:** The superficial part of triangular recess—vestibulum.

		Aa	Fa	FA	Ventricular aperture
N	Valid	100	100	100	100
	Missing	0	0	0	0
Mean		6.09	6.19^a^	6.24^b, c^	16.42
Median		6.14	6.18	6.20	16.20
Std. Deviation		0.51	0.32	0.31	1.41
Variance		0.259	0.101	0.095	1.99
Skewness		−0.554	0.154	0.095	−0.019
Std. Error of Skewness		0.241	0.241	0.241	0.241
Minimum		4.71	5.53	5.57	13.05
Maximum		7.19	6.88	6.94	20.12
Percentiles	25	5.84	5.93	6.00	15.49
	50	6.14	6.18	6.20	16.20
	75	6.45	6.46	6.51	17.44

Descriptive statistics of triangular recess aperture (mm) and ventricular aperture (Heron’s formula). Paired Samples T test between triangular recess aperture ^a^*p* < 0.001, ^b^*p* < 0.05, and ^c^*p* > 0.05.

**Figure 5:**
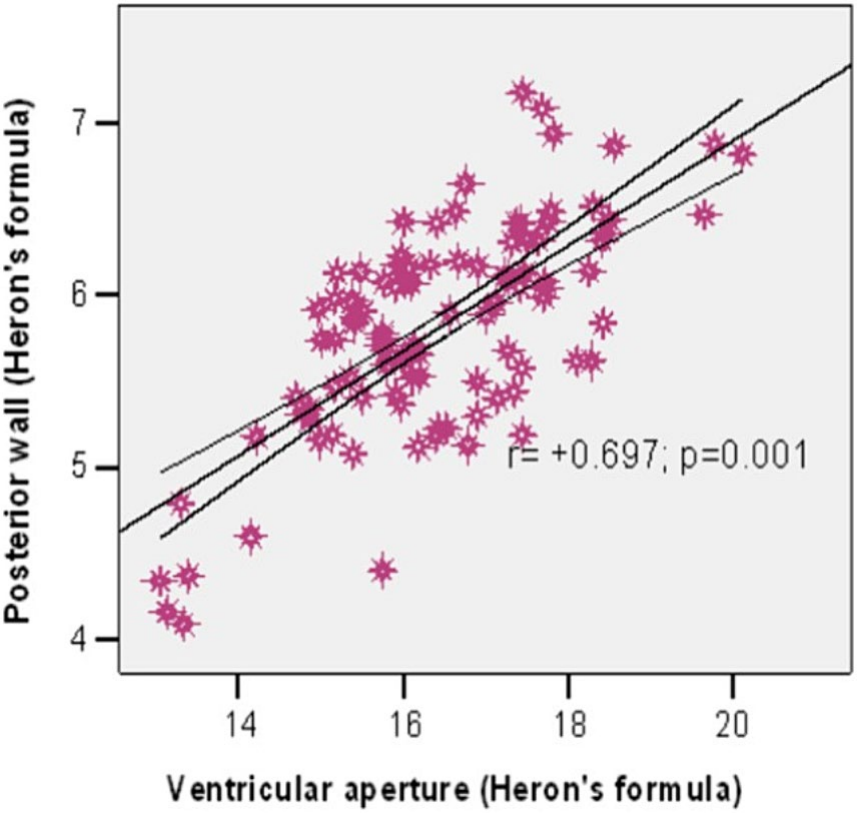
Correlation between posterior wall and ventricular aperture (Heron’s formula).

We used the Pearson correlation to investigate the association between the ventricular aperture (Heron’s formula) and the area of the posterior wall (Heron’s formula). The results show a significant strong positive correlation between them (r = +0.697, *p* = 0.001) (Figure 5), meaning that a larger ventricular aperture tends to be associated with a large posterior wall.

The Pars profunda semiperimeter (S1) varied from 4.80 to 6.13 mm, registering an average level of 5.50 mm ± 0.27, which is close to the median value (5.53 mm). The ventricular aperture semiperimeter (S2) varied from 8.30 to 10.23 mm, registering an average level of 9.26 mm ± 0.38, which is close to the median value (9.22 mm). The results of the Skewness test suggest the homogeneity of the range of values of the semiperimeters (Table 2). The average Pars profunda semiperimeter was significantly smaller than the average Ventricular aperture semiperimeter (5.50 vs. 9.26 mm; *p* = 0.001).

The Pars profunda area (Area1) varied from 16.76 to 51.58 mm^2^, registering an average level of 34.07 mm^2^ ± 7.01, which is close to the median value (34.32 mm^2^). The ventricular aperture area (Area2) varied from 170.34 to 404.72 mm^2^, registering an average level of 271.43 mm^2^ ± 46.36, which is close to the median value (262.37 mm^2^). The results of the Skewness test suggest the homogeneity of the range of values of the areas (Table 2). The average Pars profunda area was significantly lower than the average Ventricular aperture area (34.07 vs. 217.43 mm^2^; p = 0.001).

## 4 Discussion

By its position, the third cerebral ventricle represents the central segment of the ventricular system. His anterior wall contains commissural structures such as the anterior pillars of the fornix and the anterior white commissure to which the *lamina terminalis* is added. The main recess of the region is undoubtedly the triangular fossa. It stands out both by its position, which is adjacent to the interventricular foramen of Monroe, and by its clinical implications. Its limits are as follows: laterally by the anterior columns of the fornix, inferiorly by the anterior white commissure and in depth by the *lamina terminalis* (Fernandes-Silva et al., 2021; Theologou et al., 2023).

The lateral wall of the third ventricle is formed by the medial aspect of the thalamic nucleus and, respectively, by the superior aspect of the hypothalamus, between which we can identify the hypothalamic groove (Fernandes-Silva et al., 2021; Abdala-Vargas et al., 2022). The interthalamic adhesion represents a commissural structure positioned between the medial faces of the thalamic nuclei. This structure projects into the third cerebral ventricle, constituting a landmark but also an obstruction in the path of the neuroendoscope. It is inconstant and is missing in 4–30% of healthy subjects (Trzesniak et al., 2011; Damle et al., 2017; Borghei et al., 2020). In his meta-analysis, Asghar et al. (2023) state that the absence of interthalamic adhesion is associated with neuropsychiatric diseases in no less than 28.76% of these patients.

The supraoptic recess, the optic chiasm, the pituitary recess, the tuber cinereum and the mammary bodies represent the specific features of the floor, also called the infundibulum because of its shape (Fernandes-Silva et al., 2021; Abdala-Vargas et al., 2022; Theologou et al., 2023).

At the level of the posterior wall, we find the midbrain with the aqueduct of Sylvius, the posterior white commissure, as well as the pineal gland that continues towards the third ventricle’s roof with the habenular trigone (Abdala-Vargas et al., 2022; Dang et al., 2023).

The roof of the third cerebral ventricle is mostly formed by the fornix together with the velum interpositum, and in the anterior part there is communication with the lateral ventricles – the foramina of Monroe. All these structures have important functions in the activity of the central nervous system and, for this reason, they are considered critical structures (Fernandes-Silva et al., 2021; Abdala-Vargas et al., 2022; Karadag et al., 2022; Theologou et al., 2023).

Matteo Realdo Colombo originally described the triangular fossa, in 1559, as a small vertical slit, looking lubricated, and hence he called it vulva cerebri; posteriorly, it continues with a groove (of Colombo) that leads to an orifice – anus cerebri, now called hypothalamic sulcus and rostral aperture of the cerebral aqueduct, respectively. Monro (1783) described the vulva cerebri as a small triangular recess bounded laterally by the divergent columns of the fornix and inferiorly by the anterior commissure. It lies on the anterior wall of the third ventricle. Later, Schwalbe (1891) prefers the term triangular recess because of his puritanical views. In the era of routine endoscopic exploration of the ventricular system of the brain, this space returns into the limelight as a landmark during endoventricular navigation, being the first structure that appears in the endoscopic field of view.

Some authors like Longatti et al. (2003) note that it is difficult to observe and recognize the triangular recess via Monro’s foramen due to its deep position and the insertion of the pellucid septum at the dorsal aspect of the fornix columns. However, in adequate condition we can have a full view of the triangular recess. For example, the subcortical atrophy is characterized by obvious ventricular dilatations of the horns of the lateral ventricles, but also by the association with a wide interventricular foramen. In such patients, the recessus triangularis is easily seen via the lateral ventricle (Longatti et al., 2003). Feletti et al. (2016) state that in case of monoventricular dilatation and moderate hydrocephalus, the two pillars of the fornix are getting further apart, and the triangular recess becomes more visible. From a morphogenetic point of view, the triangular recess, as an anterior extension of the third ventricle, represents the frontal end of the forebrain vesicle that begins to develop telencephalic blisters (Fernandes-Silva et al., 2021; Abdala-Vargas et al., 2022; Asghar et al., 2023).

The endoscopic ventricular navigation uses landmarks to orientate the field of view inside the ventricular system. Because of its central position on the anterior aspect of the third ventricle and the close relations with Monro’s foramens, the triangular recess is the most important landmark in the region. During the endoscopic approach, it appears immediately above the anterior white commissure through the roof of the third ventricle. In MRI meatal-orbital sections, the fornico-comisural complex is also visible (Longatti et al., 2003; Karadag et al., 2022).

The endoscopic procedures involving cerebral ventricular system require detailed knowledge of the morphological aspect of the endoluminal ventricular system. For a successful surgery, the anatomical details are important as they guide the surgeon towards the injury, and help him locate and safely remove the pathological process. In order to achieve this, structures such as the Monro’s hiatus, commisura rostralis, recessus supraopticus, recessus infundibuli or commisura caudalis represent landmarks for the intraventricular navigation (Theologou et al., 2023). From the point of view of endoscopic procedures, the use of the endoscopic extended transforaminal approach has gained ground in front of the classic transchoroidal approach. Both techniques highlight the structures of the third cerebral ventricle, but the first one is a conservative technique in relation to the septal vein and the fornix (Jean et al., 2019; Cerro Larrazabal et al., 2023; Narro-Donate et al., 2023). Tawk et al. (2020) describe an innovative technique that increases the mobility of the endoscope by opening the choroidal fissure. In this way, the antero-posterior diameter of the foramen of Monroe increases, allowing a better visualization of the structures of the III ventricle and, of course, the approach to the detected formations.

Numerous anatomists dispute the existence of the triangular recess (Standring, 2005), while others mention it as an inconstant structure (Friede, 1961). However, as Mortazavi et al. (2014) mentioned in their paper, we identified the triangular recess in all dissected brains.

The length of the intercrural rostral commissure and the aria of the ventricular aperture are important morphometric parameters for the translaminar ventriculoscopy. This procedure involves the creation of a *lamina terminalis* stoma that allows the flexible or rigid endoscope to penetrate the third ventricle (Vulcu et al., 2014). The position of the triangular recess, adjacent to the foramen of Monro, makes it a good candidate access point to the lateral ventricle’s endoscopic procedures.

This morphologic and morphometric evaluation of the triangular fossa could be an important tool for the assessment of the hydrocephalous, considering that the morphology and the morphometric parameters alter in this syndrome (Ciappetta et al., 2019). The endoscopic procedures tend to be the first therapeutic choice for obstructive ventricular syndromes. The evaluation of the success or failure of this type of procedure has sparked many controversies. Nevertheless, it is unanimously accepted that the evaluation can be carried out 3–6 months after the intervention. While some neurosurgeons prefer the morphometric assessment of the width of the third cerebral ventricle, others consider the measurement of the CSF flow as being more faithful (Wilcock et al., 1997). Instead, Azab et al. (2015) demonstrated in his study the early modification of the opening angle of the infundibular recess after endoscopic ventriculostomy. This observation imposes the role of the structures of the anterior-inferior wall of the III ventricle in the evaluation of the hydrocephalus and the therapeutic effect. In this context, our study achieves an anatomical norm of the structures that represent the triangular fossa. We argue that it is a complex structure having two distinct segments which we call *pars profunda* and *pars superficialis*. In addition, the morphometric analysis demonstrated that it is a structure that is more tall than wide, the base of the deep part CC (3.54 mm ± 0.37) being statistically smaller than the sides FC (3.75 mm ± 0.11) and Fc (3.72 mm ± 0.11) (*p* = 0.001). This characteristic is also maintained in the case of the superficial part, segment Aa being statistically smaller than segment FA (*p* = 0.011), but not compared to segment Fa (*p* = 0.115). However, the defining element remains the area of communication between the pars profunda and pars superficialis, respectively between the pars superficialis and the III ventricle. The pressure changes from hydrocephalus determine the separation of the two anterior columns of the fornix and implicitly the increase of the surface of these communications. The anatomical norm determined by us is 34.07 mm^2^ ± 7.01 for the *pars profunda—pars superficialis* communication area, respectively 271.43 mm^2^ ± 46.36 for the ventricular opening. The area of the deep part aperture is significantly smaller than the area of the ventricular aperture, the difference being statistically significant (*p* = 0.001).

As an initial landmark for the anterior edge of the third cerebral ventricle, the triangular recess is the first to appear in the endoscopic field of view, and therefore, a study that correlates the morphometric parameters to the degree of hydrocephalic enlargement has to be done.

The recessus triangularis is a structure which is difficult to be revealed on the MRI images due to its deep and oblique position. However, it is possible to highlight the commissuro-fornical complex that includes the triangular recess, rostral commissura and the cruses of the fornix, in a meatal-orbital section, using the regression forests method (Liu and Dawant, 2015). In consequence, the 3D-constructive inference in steady state (3D CISS) MRI sequence can identify the landmarks of the third ventricle, especially the triangular recess, in order to quantify the degree of enlargement. This pre-surgical analysis enables a choice to be made between the endoscopic third ventriculostomy and the ventriculoperitoneal shunt for patients with obstructive hydrocephalus (Takahashi, 2006; Shi et al., 2013).

Our study is subject to several limitations that are important to acknowledge. Firstly, it is a study carried out on anatomical parts preserved in 10% formalin solution. This causes structural changes in the nervous tissue with increased hardness, which is a favorable effect during dissections, but at the same time it causes a degree of dehydration. To counteract this effect, we performed a minimum preservation of only 10 weeks for each sample. Secondly, the measurement method can represent a source of error. To compensate for these errors, we performed extensive dissections and placed the millimeter scale as close as possible and mandatorily in the plane of the measured structure. Despite these limitations, we consider that our study demonstrates the landmark value of the triangular fossa in intraventricular endoscopic interventions.

## 5 Conclusion

The triangular recess represents the anterior extension of the third ventricle and the primitive cavity of the neural tube. Our study enabled us to imagine an original model of organization in a three-dimensional form. It consists of a deep part that is in intimate contact with *lamina terminalis* and a superficial part that communicates posteriorly with the third cerebral ventricle and inferiorly with the subcommissural recess. Despite the limitations, the measurements establish an anatomical morphometric norm of the triangular recess. We stress therefore the importance of the triangular recess as a landmark in the frontal endoscopic approach to the third cerebral ventricle, in assessing the degree of hydrocephalus and the therapeutic effect of endoscopic ventriculostomy.

## Data availability statement

The raw data supporting the conclusions of this article will be made available by the authors, without undue reservation.

## Author contributions

AN: Conceptualization, Investigation, Supervision, Writing – original draft. VL: Formal analysis, Project administration, Writing – review & editing. AL: Formal analysis, Visualization, Writing – review & editing. RT: Conceptualization, Investigation, Writing – original draft. II: Data curation, Resources, Writing – original draft. CS: Investigation, Methodology, Project administration, Writing – review & editing. SV: Methodology, Visualization, Writing – review & editing. AH: Investigation, Software, Writing – original draft. GS: Software, Visualization, Writing – original draft. MU: Validation, Writing – original draft. CD: Data curation, Resources, Writing – review & editing. CT: Methodology, Supervision, Validation, Writing – original draft.

## Glossary

TR: Triangular recess
Pars profunda: Cc – upper edge of the anterior white commissure included between the columns of the fornix; Fc – the right anterior columns of the fornix included between the tip of the fornix and the upper edge of the anterior white commissure; FC – the left anterior columns of the fornix included between the tip of the fornix and the upper edge of the anterior white commissure.
Pars superficialis: Aa – lower edge of the anterior white commissure included between the columns of the fornix; Fa – the right anterior columns of the fornix included between the tip of the fornix and the lower edge of the anterior white commissure; FA – the left anterior columns of the fornix included between the tip of the fornix and the lower edge of the anterior white commissure.

## References

[ref1] Abdala-Vargas N. J. Cifuentes-Lobelo H. A. Ordoñez-Rubiano E. Patiño-Gomez J. G. Villalonga J. F. Lucifero A. G.. (2022). Anatomic variations of the floor of the third ventricle: surgical implications for endoscopic third ventriculostomy. Surg. Neurol. Int. 27:218. doi: 10.25259/SNI_404_2022 PMC9168335 35673649

[ref2] Asghar A. Narayan R. K. Kumar P. Ravi K. S. Tubbs R. S. Patra A.. (2023). Absence of the interthalamic adhesion (ITA) as a neuroanatomical association or risk factor for neuropsychiatric disorders: a systemic review and meta-analysis. Indian J. Psychiatry 65, 985–994. doi: 10.4103/indianjpsychiatry.indianjpsychiatry_744_22, PMID: 38108053 PMC10725209

[ref3] Azab W. A. Mijalcic R. M. Abdelnabi E. A. Khan T. A. Mohammad M. H. Shaat M. S. (2015). Infundibular recess angle reduction after endoscopic third ventriculostomy: does it reflect clinical success? World Neurosurg. 84, 549–554. doi: 10.1016/j.wneu.2015.04.007, PMID: 25871782

[ref4] Borghei A. Cothran T. Brahimaj B. Sani S. (2020). Role of Massa intermedia in human neurocognitive processing. Brain Struct. Funct. 225, 985–993. doi: 10.1007/s00429-020-02050-5, PMID: 32124014

[ref5] Cerro Larrazabal L. Ibáñez Botella G. Ros Sanjuán Á. Ros López B. Iglesias Moroño S. Arráez Sánchez M. Á. (2023). Neuroendoscopic transventricular transchoroidal approach for access to the posterior zone of the third ventricle or pineal region. Neurosurg. Rev. 46:323. doi: 10.1007/s10143-023-02210-1 38041741

[ref6] Ciappetta P. Tropeano M. P. Gitto L. Pescatori L. (2019). Schwalbe's triangular fossa: normal and pathologic anatomy on frozen cadavers. Anatomo-magnetic resonance imaging comparison and surgical implications in colloid cyst surgery. World Neurosurg. 128, e116–e128. doi: 10.1016/j.wneu.2019.04.058, PMID: 30981795

[ref7] Damle N. R. Ikuta T. John M. Peters B. D. DeRosse P. Malhotra A. K.. (2017). Relationship among interthalamic adhesion size, thalamic anatomy and neuropsychological functions in healthy volunteers. Brain Struct. Funct. 222, 2183–2192. doi: 10.1007/s00429-016-1334-6, PMID: 27866270 PMC5816973

[ref8] Dang D. D. Rechberger J. S. Leonel L. C. P. C. Rindler R. S. Nesvick C. L. Graepel S.. (2023). Anatomical step-by-step dissection of common approaches to the third ventricle for trainees: surgical anatomy of the anterior transcortical and interhemispheric transcallosal approaches, surgical principles, and illustrative pediatric cases. Acta Neurochir. 165, 2421–2434. doi: 10.1007/s00701-023-05697-1, PMID: 37418043

[ref9] Dumitrescu A. M. Eva L. Haba D. Cucu A. I. Dumitrescu G. F. Burduloi V. M.. (2022). Anatomical study of circle of Willis on fresh autopsied brains. A study of a Romanian population. Romanian J. Morphol. Embryol. 63, 395–406. doi: 10.47162/RJME.63.2.10, PMID: 36374144 PMC9804071

[ref10] Feletti A. Alicandri-Ciufelli M. Pavesi G. (2016). Transaqueductal trans-Magendie fenestration of arachnoid cyst in the posterior fossa. Acta Neurochir. 158, 655–662. doi: 10.1007/s00701-016-2734-3, PMID: 26883551

[ref11] Fernandes-Silva J. Silva S. M. Alves H. Andrade J. P. Arantes M. (2021). Neurosurgical anatomy of the floor of the third ventricle and related vascular structures. Surg. Radiol. Anat. 43, 1915–1925. doi: 10.1007/s00276-021-02785-8, PMID: 34128100

[ref12] Friede R. (1961). Surface structures of the aqueduct and the ventricular walls. J. Comp. Neurol. 116, 229–247. doi: 10.1002/cne.901160210 13701951

[ref14] Jean W. C. Tai A. X. Hogan E. Herur-Raman A. Felbaum D. R. Leonardo J.. (2019). An anatomical study of the foramen of Monro: implications in management of pineal tumors presenting with hydrocephalus. Acta Neurochir. 161, 975–983. doi: 10.1007/s00701-019-03887-4, PMID: 30953154

[ref15] Karadag A. Camlar M. Turkis O. F. Bayramli N. Middlebrooks E. H. Tanriover N. (2022). Endoscopic Endonasal approach to the third ventricle using the surgical corridor of the reverse third Ventriculostomy: Anatomo-surgical nuances. J. Neurol. Surg. B Skull Base 84, 96–306. doi: 10.1055/a-1808-1359 PMC10171930 37187474

[ref16] Liu Y. Dawant B. M. (2015). Automatic localization of the anterior commissure, posterior commissure, and midsagittal plane in MRI scans using regression forests. J. Biomed. Health Inform. 19, 1362–1374. doi: 10.1109/JBHI.2015.2428672, PMID: 25955855 PMC4519399

[ref17] Longatti P. Martinuzzi A. Fiorindi A. Malobabic S. Carteri A. (2003). Endoscopic anatomic features of the triangular recess. Neurosurgery 52, 1491–1494. doi: 10.1227/01.NEU.0000065184.29846.E0, PMID: 12762898

[ref18] Majmundar N. Quillin J. Liu J. K. Agarwalla P. K. (2023). Bilateral infraoptic origin of the anterior cerebral artery: illustrative case. J. Neurosurg. Case Lessons. 6:CASE23418. doi: 10.3171/CASE23418 37956417 PMC10651390

[ref19] Makowicz G. Poniatowska R. Lusawa M. (2013). Variants of cerebral arteries – anterior circulation. Pol. J. Radiol. 78, 42–47. doi: 10.12659/PJR.889403, PMID: 24115959 PMC3789932

[ref20] Monro A. (1783). Observations on the structure and functions of the nervous system. Edinburgh: Creech & Johnson.

[ref21] Mortazavi M. M. Adeeb N. Griessenauer C. J. Sheikh H. Shahidi S. Tubbs R. I.. (2014). The ventricular system of the brain: a comprehensive review of its history, anatomy, histology, embryology, and surgical considerations. Childs Nerv. Syst. 30, 19–35. doi: 10.1007/s00381-013-2321-3, PMID: 24240520

[ref22] Narro-Donate J. M. Guil-Ibañez J. J. Castelló-Ruiz M. J. García-Pérez F. Urreta-Juarez G. Masegosa-González J. (2023). Endoscopic extended transforaminal approach (medial subchoroid) as an alternative to the classical transchoroidal approach: technical note. J. Clin. Neurosci. 116, 39–43. doi: 10.1016/j.jocn.2023.08.015, PMID: 37611370

[ref23] Nedelcu A. H. Ţepordei R. T. Sava A. Stan C. I. Aignătoaei A. M. Ţăranu T.. (2016). Supernumerary fronto-orbital arteries arising from contralateral anterior cerebral artery associated with partially duplicated anterior communicating artery – case study and literature review. Romanian J. Morphol. Embryol. 57, 1159–1163, PMID: 28002539

[ref24] Schroeder H. W. Niendorf W. R. Gaab M. R. (2002). Complications of endoscopic third ventriculostomy. J. Neurosurg. 96, 1032–1040. doi: 10.3171/jns.2002.96.6.1032 12066903

[ref25] Schwalbe G. A. (1891). Morphologische Arbeiten. Jena: G. Fischer.

[ref26] Shi J. Fu W. Wu Q. Zhang H. Zheng Z. Zhu J. (2013). Endoscopic third ventriculostomy associated 3D-construcive inference steady state MRI for obstructed hydrocephalus: a retrospective study. Clin. Neurol. Neurosurg. 115, 896–901. doi: 10.1016/j.clineuro.2012.08.038, PMID: 23026416

[ref13] Standring S. (2005). Gray’s anatomy. The anatomical basis of clinical practice. 39th ed. London: Elsevier Churchill Livingstone, 227–418.

[ref27] Takahashi Y. (2006). Long-term outcome and neurologic development after endoscopic third ventriculostomy versus shunting during infancy. Childs Nerv. Syst. 22, 1591–1602. doi: 10.1007/s00381-006-0190-8, PMID: 17021728

[ref28] Tawk R. G. Akinduro O. O. Grewal S. S. Brasiliense L. Grand W. Grotenhuis A. (2020). Endoscopic transforaminal transchoroidal approach to the third ventricle for cystic and solid tumors. World Neurosurg. 134, e453–e459. doi: 10.1016/j.wneu.2019.10.099, PMID: 31669244

[ref29] Theologou M. Kouskouras K. Natsis K. Varoutis P. Zaggelidou E. Tsonidis C. (2023). Microanatomic morphometric characteristics of the third ventricle floor. Brain Sci. 13:580. doi: 10.3390/brainsci13040580, PMID: 37190545 PMC10136925

[ref30] Trzesniak C. Kempton M. J. Busatto G. F. de Oliveira I. R. Galvão-de Almeida A. Kambeitz J.. (2011). Adhesio interthalamica alterations in schizophrenia spectrum disorders: a systematic review and meta-analysis. Prog. Neuro-Psychopharmacol. Biol. Psychiatry 35, 877–886. doi: 10.1016/j.pnpbp.2010.12.024, PMID: 21300129

[ref31] Vulcu S. Tschabitscher M. Mueller-Forell W. Oertel J. (2014). Transventricular fenestration of the lamina terminalis: the value of a flexible endoscope: technical note. J. Neurol. Surg. A Cent. Eur. Neurosurg. 75, 207–216. doi: 10.1055/s-0033-1345684, PMID: 23939681

[ref32] Wilcock D. J. Jaspan T. Worthington B. S. Punt J. (1997). Neuroendoscopic third ventriculostomy: evaluation with magnetic resonance imaging. Clin. Radiol. 52, 50–54. doi: 10.1016/S0009-9260(97)80306-6, PMID: 9022581

